# Facilitators of and Barriers to Lifestyle Support and eHealth Solutions: Interview Study Among Health Care Professionals Working in Cardiac Care

**DOI:** 10.2196/25646

**Published:** 2021-10-15

**Authors:** Talia R Cohen Rodrigues, David R de Buisonjé, Mike Keesman, Thomas Reijnders, Jessica E van der Geer, Veronica R Janssen, Roderik A Kraaijenhagen, Douwe E Atsma, Andrea W M Evers

**Affiliations:** 1 Health, Medical, and Neuropsychology Unit Faculty of Social and Behavioural Sciences Leiden University Leiden Netherlands; 2 Department of Human-Centered Design Faculty of Industrial Design Engineering Technical University of Delft Delft Netherlands; 3 Department of Cardiology Leiden University Medical Center Leiden Netherlands; 4 NDDO Institute for Prevention and Early Diagnostics (NIPED) Amsterdam Netherlands; 5 Vital10 Amsterdam Netherlands; 6 Department of Psychiatry Leiden University Medical Center Leiden Netherlands; 7 Medical Delta Leiden-Delft-Erasmus Universities Delft Netherlands

**Keywords:** eHealth, digital health, cardiovascular disease, cardiac care, lifestyle change, lifestyle support, intervention, health care professionals, implementation, interview, facilitators, barriers

## Abstract

**Background:**

Cardiovascular diseases (CVDs) pose a significant health threat and reduce both people’s life expectancy and quality of life. Healthy living is a key component in the effective prevention and treatment of CVD. However, health care professionals (HCPs) experience difficulties in supporting lifestyle changes among their patients. eHealth can provide a solution to these barriers.

**Objective:**

This study aims to provide insights into the factors HCPs find important in the support of patients with CVD in the uptake of and adherence to a healthy lifestyle and the perceived facilitators of and barriers to using eHealth to provide lifestyle support to patients with CVD.

**Methods:**

In-depth interviews were conducted with 16 Dutch HCPs specializing in lifestyle support in cardiac care.

**Results:**

We identified 13 themes, of which the first 12 concerned lifestyle support in general and were related to intervention, patient, or health care. Throughout these themes, the use of eHealth reoccurred as a potential facilitator of or solution to barriers to lifestyle support. Our final theme specifically concerned barriers to the adoption and usability of eHealth.

**Conclusions:**

HCPs do recognize the potential advantages of eHealth while experiencing barriers to using digital tools. Incorporating their needs and values in the development of lifestyle support programs, especially eHealth, could increase their use and lead to a more widespread adoption of eHealth into health care.

## Introduction

### Background

Cardiovascular diseases (CVDs) are the most common cause of death globally [1] and drastically reduce the quality of life [2]. Most CVDs can be prevented and treated by addressing behavioral risk factors such as smoking, poor diet, physical inactivity, and low sleep quality [3,4]. Lifestyle interventions for patients with CVD have been shown to improve risk factors and decrease cardiac readmissions and mortality [5] and are therefore recommended by national and international guidelines on cardiac prevention and rehabilitation [3,6]. Furthermore, a healthy lifestyle has mortality-reducing effects comparable with those of medication intake [7].

Notwithstanding these advantages, health care professionals (HCPs) seem to be hesitant to discuss—let alone prescribe—lifestyle interventions to their patients [8]. For instance, studies among Dutch general practitioners found that they are generally unlikely to mention CVD risk factors, such as lifestyle, during their consultations [9] and only provided advice concerning healthy living in 1 out of 6 consultations to people with hypertension complaints [10]. In addition, only 1 out of 5 primary care physicians indicated that European CVD guidelines concerning lifestyle were being implemented [11]. In line with this, most patients with CVD have an unhealthy lifestyle [12]. A number of barriers have been identified as possible explanations for the low attention paid to lifestyle changes and their respective programs. HCPs have mentioned a low degree of patient motivation, a lack of knowledge about or experience with providing lifestyle advice, insufficient time during consultations, a lack of financial incentives, little external options to refer their patients to, and HCPs’ perception that health promotion is ineffective in CVD prevention and treatment [11,13-16]. These barriers could possibly explain why only half of the patients with CVD are actually offered lifestyle modification programs after discharge [17]. To increase the uptake of and adherence to lifestyle interventions among patients with CVD, it is crucial to consider HCPs’ needs and barriers.

A potential solution to overcome these barriers could be the increased use of digital tools to provide automated or remote support, which can incorporate interactive web-based components and digital wearables for home measurement, known as eHealth [18]. Recent studies have shown that eHealth can be effective in the prevention and treatment of noncommunicable diseases such as CVD [19,20]. Despite these promising results, the acceptance and successful implementation of digital tools in health care is low because of the barriers that HCPs experience [21-23]. To design digital interventions that have an impact, human and contextual factors should be taken into account, including the needs and values of stakeholders such as HCPs [24,25]. Interview studies uncovering HCPs’ views on lifestyle interventions and their own role in health promotion among their patients [15,26-29] and on the use of eHealth in lifestyle support [30-34] have been conducted before. However, to our knowledge, no studies have mapped out the attitudes toward both lifestyle support and eHealth of HCPs specialized in CVD specifically. Importantly, given that the effectiveness and needs related to lifestyle support and eHealth are highly context dependent [24], specific knowledge is needed in the context of cardiac care.

### Objective

This study aims to gain insight into the facilitators and barriers that HCPs specialize in cardiac care experience in lifestyle support for the prevention and treatment of CVD and to investigate their views on eHealth tools. We performed in-depth interviews with HCPs to answer two main questions: (1) What factors are important in supporting CVD patients in the uptake of and adherence to a healthy lifestyle? (2) What are the (potential) facilitators of and barriers to eHealth tools in providing lifestyle support to patients with CVD?

## Methods

### Sample

We interviewed 16 Dutch HCPs (10 women) specializing in supporting patients with CVD and with experience in lifestyle change. To ensure a diverse and representative collection of perspectives, we included professionals with varying backgrounds from multiple institutions located in different parts of the Netherlands (Table 1). Professionals were selected based on eHealth affinity within the department or organization they worked in and asked how they applied eHealth in their own job to verify some level of eHealth experience. In line with these guidelines, 16 interviews would be sufficient for information saturation [35].

**Table 1:** Organization and professional background of respondents (N=16).

Organization and professional background		Respondents, n (%)
**Academic hospital A**		
	Nurse practitioner working in cardiac rehabilitation	2 (12)
**Academic hospital B**		
	Neurovascular nurse practitioner	1 (6)
	Physician assistant specialized in cardiovascular risk factor management	1 (6)
**Hospital A**		
	Physiotherapist working in cardiac rehabilitation	1 (6)
	Nurse practitioner working in cardiac rehabilitation	1 (6)
**Hospital B**		
	Physician-researcher working in cardiac rehabilitation	1 (6)
	Nurse practitioner working in cardiac rehabilitation	1 (6)
**Hospital C**		
	Neurologist specialized in cardiac rehabilitation	1 (6)
	Nurse practitioner working in cardiac rehabilitation	1 (6)
**Cardiac rehabilitation center A**		
	Cardiologist in residence	1 (6)
	Lifestyle coach working in cardiac rehabilitation	1 (6)
**Cardiac rehabilitation center B**		
	Physiotherapist working in cardiac rehabilitation	1 (6)
**Cardiac rehabilitation center C**		
	Psychologist specialized in cardiac rehabilitation	1 (6)
**General practice center A**		
	General practitioner specialized in CVD^a^ care	1 (6)
	Nurse practitioner working in cardiac rehabilitation	1 (6)

^a^CVD: cardiovascular disease.

### Procedure

We used convenience sampling and approached organizations within the network of care partners. We asked for professionals within the organization who were most directly involved with lifestyle support of patients with CVD, whom we sent an email with general information about the research goals. After the HCP expressed willingness to cooperate, interview appointments were made by phone and performed at the interviewee’s preferred location. Before the start of the interview, information about the research project and the goals and methods of the interview were provided. The interviewee signed the informed consent form, after which the voice recording and interview started. No (financial) compensation was offered to participate.

The interviews were conducted in Dutch, between November 2017 and February 2018, and took 45-90 minutes. One researcher led the interview, whereas another took notes, and roles were alternated between each interview (DRDB and JEVDG). The voice recordings were transcribed and pseudonymized to secure anonymity (Brecht Otto and Pauline van Wolferen). We used a general interview guide approach, as the interviews were based on a semistructured list of questions that allowed for further elaboration based on answers. The questions were divided into six topics (Textbox 1). We asked about both the use of eHealth (digital tools to provide automated or remote support with interactive web-based components) and wearables and sensors (eg, pedometers). Interview topics were defined based on the research questions and assessed whether they would provide answers to these questions. We also included questions about the interviewees’ profiles (eg, job description and experience with eHealth). This study only discusses the data from interview topics 1-5, which are relevant to our specific research questions. The data regarding the sixth interview topic (Reward Program to Promote Healthy Living) do not belong to the scope of this study and are used in another publication.

**Interview guide.**

**Facilitating and impeding factors in the uptake of and adherence to a healthy lifestyle for patients with cardiovascular disease**

- What do cardiovascular disease patients need to do in their home environment to achieve sustainable lifestyle change?
- What things that seem to work well for cardiovascular disease patients in changing their lifestyle?
- What impedes cardiovascular disease patients in changing their lifestyle?
- What solutions do cardiovascular disease patients have for these barriers?

**Facilitating and impeding factors in providing lifestyle support to patients with cardiovascular disease**

- How do you provide lifestyle support to cardiovascular disease patients?
- What works well in providing lifestyle support?
- What impedes providing lifestyle support?
- What solutions do you have for these barriers?

**Stakeholders involved in providing lifestyle support to patients with cardiovascular disease**

- What do you, as a health care professional, need to better provide lifestyle support to cardiovascular disease patients?
- With whom do you cooperate in providing lifestyle support to cardiovascular disease patients?

**Facilitating and impeding factors in using eHealth to provide lifestyle support to patients with cardiovascular disease**

- What things go well in your use of eHealth to provide lifestyle support to cardiovascular disease patients?
- What impedes your use of eHealth to provide lifestyle support to cardiovascular disease patients?
- What solutions do you have for these barriers?
- What do you, as a health care professional, need to better make use of eHealth to provide lifestyle support to cardiovascular disease patients?

**Facilitating and impeding factors in using wearables and sensors to provide lifestyle support to patients with cardiovascular disease**

- To what extent do you use wearables and sensors to provide lifestyle support to cardiovascular disease patients?
- What things go well in your use of wearables and sensors to provide lifestyle support to cardiovascular disease patients?
- What impedes your use of wearables and sensors to provide lifestyle support to cardiovascular disease patients?
- What solutions do you have for these barriers?

### Analyses

The transcripts were sorted into meaningful clusters based on a content analysis approach to ensure that insights emerged based on the data [36]. Relevant pieces of data were retrieved from the text and coded and categorized into themes. For each of the transcripts, 2 researchers (DRDB and JEVDG) independently marked quotations in Microsoft Word containing relevant information. These quotations were compared, and a consensus document for each transcript was created. The quotations were transferred to Microsoft Excel, coded in a separate column to allow for interpretation, color coded to indicate whether the quotation was related to eHealth, and subsequently categorized into themes (TRCR). In discussion with a second researcher (MK), a definitive set of 13 themes emerged on which each of the quotations were fit (Textbox 2). Consensus with an independent coder (Magali de Rooy) was reached at once, with an interrater agreement of 74% and sufficient interrater reliability (Krippendorff α=.697), which indicated that the developed list of themes adequately represented the structure of the data. Quotation examples in text were translated into English by 2 researchers (TRCR and DRDB).

**Identified themes after coding.**

**Intervention-related factors**

1. Autonomy

- Factors that concern the extent to which the patient has the freedom to make decisions about lifestyle change for themselves
- Quotations that concern the feeling of control in the process, the amount of self-determination, and insight into one’s own health

2. Goal setting

- Factors that are related to setting goals in lifestyle change
- Quotations concerning the quantity, content, and design of these goals

3. Personalization

- Factors that are related to the adjustment of a healthy lifestyle and revalidation program to the needs and wishes of the patient
- Quotations that concern the personal relevance, feasibility, and attractiveness of the revalidation process

**Patient-related factors**

4. Motivation

- Factors that facilitate or impede the willpower to start or maintain lifestyle change
- Quotations that concern the extent to which patients are willing to work on their lifestyle and their intrinsic and extrinsic motivation

5. Condition of the patient

- Physical, mental, or cognitive impairments that impede the patient in the uptake of and adherence to a healthy lifestyle (eg, pain, depression, stress, addictions, and age)
- Both conditions that already existed and those because of their illness

6. Psychological characteristics

- Characteristics and traits of the patient that facilitate or impede the uptake of and adherence to a healthy lifestyle
- Quotations that concern personality or personal predispositions of the patient (eg, self-efficacy, resistance, and sense of responsibility)

7. Environmental factors

- Factors in the home environment and daily life of the patient that facilitate or impede the uptake of and adherence to a healthy lifestyle
- Quotations that concern the direct surroundings of the patient, which one cannot control (difficult domestic situations, socioeconomic status, and access to health or unhealthy options)

8. Social network

- Factors in the social circle of the patient that facilitate or impede the uptake of and adherence to a healthy lifestyle
- Quotations that concern the role of friends, family, and acquaintances in the patient’s lifestyle

**Health care–related factors**

9. Format of professional support

- Factors that determine the way in which support of the patient is shaped and structured and facilitate or impede the uptake of and adherence to a healthy lifestyle
- Quotations that concern the implementation, frequency, and format of support

10. Relationship with the patient

- Factors that are related to the personal relationship between health care professional and patient
- Quotations that indicate the way in which such a relationship is established and what it should entail

11. Continuity of professional support

- Factors that are related to long-term support of the patient and facilitate or impede maintaining a healthy lifestyle
- Quotations that concern lifestyle change in the long run, outside the health care environment, and continuing the revalidation process by the patient

12. Organization of care

- Practical factors that influence the provided health care, both physical facilities (eg, health care professional’s practice) and nontangible influences (eg, regulations, finances) that facilitate or impede lifestyle support
- Quotations that concern the availability of care and the extent to which health care professionals’ can do their job and the way they are ought to do

**eHealth-related factors**

13. Barriers to eHealth

- Factors that are related to the implementation of eHealth (digital tools) in lifestyle support
- Quotations that concern the difficulties in using and implementation of technology and data in the current health care system

## Results

### Barriers to and Facilitators of Lifestyle Support

Of the 13 identified themes, 12 concerned lifestyle support in general (Textbox 2). The subjects of these themes were related to the intervention, the patient, or health care in general.

### Intervention-Related Factors

#### Autonomy

Nearly all (15/16, 94%) HCPs mentioned that patients need to feel a sense of ownership over their lifestyle change process instead of being just another patient undergoing rehabilitation. One HCP was especially concerned about the lack of choice in cardiac rehabilitation:

People are forced to do so many things, they end up in an obligatory trajectory. That is already quite a lot. So I think that can be a barrier.

Quote 89, HCP 8

Self-monitoring (eg, heartbeat or weight) and information about both their disease and the benefits of a healthy lifestyle were mentioned by 10 HCPs to be essential for patients to feel a sense of control. This allows them to act independently of their HCPs when they notice irregularities:

It is also important for patients that they get more insight themselves...That they can alert us whenever they are training independently and say “my heartrate shows irregularities or is not going up.”

Quote 73, HCP 1

However, 3 HCPs mentioned that self-monitoring might have the downside of becoming an obsession, as people could fixate on numbers rather than their own body.

#### Goal Setting

More than half of the HCPs (9/16, 56%) mentioned the importance of goal setting in a healthy lifestyle. Patients would reach the most success when the number of goals at a given time is limited; when the goals are formulated in a specific, measurable, acceptable, realistic, and timely way; and when the goals are personally relevant for the patient. Accomplishment of these goals provides a rewarding feeling, which increases motivation to continue:

I want them to create their own success story...I choose something

a goal] of which I guess that person will be able to achieve in the upcoming week. And that turns into motivation...[Quote 133, HCP 8

#### Personalization

Of all HCPs, 56% (9/16) experienced that a lifestyle intervention will succeed when the provided support is tailored to patients’ needs, capabilities, and preferences. For instance, for some, it is more important to work on their eating habits, whereas for others, an increase in physical activity is more relevant. At the same time, HCPs mentioned difficulties in finding out what their patients actually wanted and needed, which made it challenging to individualize the program:

And I’d really like to get to know the person on the other side of the table, what kind of information that person would like to receive. I find it hard to know: How would someone like to be motivated.

Quote 148, HCP 4

### Patient-Related Factors

#### Motivation

A key theme throughout the interviews (14/16, 88%) was the level of motivation of the patient. Intrinsic motivation was deemed essential to successfully complete—or even start—a lifestyle program. Such intrinsic motivation is not always self-evident because of low awareness about the current and future health impact of an unhealthy lifestyle. About 38% (6/16) of HCPs mentioned how the occurrence of the disease acts as the tipping point for patients to change their lifestyle:

People already know that they are unhealthy and that they should make changes. Often you will notice that such a crisis causes them to actually do so.

Quote 54, HCP 13

To maintain the level of motivation, after the initial scare from the incident has passed, 56% (9/16) of HCPs mentioned that patients need to see progress of their effort, preferably through tangible results (eg, increased performance durations). Extrinsic motivation, in the form of both material and nonphysical incentives (eg, positive feedback), was mentioned by 44% (7/16) of HCPs to play a role:

Rewards are on multiple levels, a reward can also be that you are just being noticed by your significant other, brother, sister, friend. But it can also be a more literal reward, you know, that you buy something for yourself. Or that you tell yourself, well done.

Quote 57, HCP 13

#### Condition of the Patient

Health-related issues hindering patients from initiating or maintaining a healthy lifestyle were mentioned by 75% (12/16) of HCPs. These issues are physical, cognitive, or mental and are either pre-existing or because of cardiac incidents. For example, reduced mobility in older patients is a physical barrier to physical activities or reaching the clinic. Frequently mentioned mental barriers were depressive symptoms and fear, such as concerns about physical capabilities after a cardiac incident:

Especially people who experience persistent heart complaints, that cause a lot of anxiety, they think: I won’t push myself. When I start exercising, I will experience it again.

Quote 177, HCP 4

#### Psychological Characteristics

Most HCPs (13/16, 81%) mentioned the role of their patients’ personalities either as facilitators or as barriers. Patients need to be disciplined, and most importantly, some level of self-awareness helps to reflect on their own behavior and acknowledge their own role in the process. Patients who come up with excuses for not performing healthy behaviors are most difficult to work with:

But there is also a big group of people who are just very resistant to change, who are mainly externalizing and say: “I can’t do anything about it.” Or who continuously come up with excuses about why things can’t change. Yes, that is the most difficult group to work with. That is also the most unhealthy group.

Quote 215, HCP 15

In addition to personality characteristics, another frequently mentioned barrier was previously developed bad habits.

#### Environmental Factors

Factors related to the daily environment of the patients were identified by 56% (9/16) of HCPs. For instance, difficult domestic situations are often given more priority and can therefore reduce the success of a healthy lifestyle initiated in the clinic. Some HCPs (4/16, 25%) explicitly stated that socioeconomic status (eg, language barriers) affects people’s lifestyles:

When it comes to handing out flyers as well, I come across situations such as: “I can’t read.” Not very frequently, but it happens every now and then.

Quote 230, HCP 10

According to 25% (4/16) of HCPs, government authorities should take responsibility for creating a healthy environment (eg, offering healthy food in hospitals, stricter tobacco and alcohol regulations, or regulating the prices of food) and providing health education.

#### Social Network

The roles of both close (family and friends) and distant others were mentioned by 50% (8/16) of HCPs. Other people function as social controls or exert some level of group pressure. A sense of cohesion through engaging in healthy activities with others is a great motivator:

...an exemplary role, sociability, a social aspect, controlling aspect, when you are part of a group people will ask about you: How are you doing, where were you? All those kind of things play a role.

Quote 246, HCP 1

The importance of the social network of patients in providing practical and psychological support was emphasized by 38% (6/16) of HCPs. However, HCPs worried that overly critical family members or friends could also negatively influence the process. A second concern was social norms, as some unhealthy behaviors (such as drinking too much alcohol) are less socially accepted and therefore more difficult for patients to be open about:

Well, the subject is more of a taboo. It is automatically an issue. When you drink too much, you are an alcoholic. Eating too much, well, that happens to all of us. That we are snacking a little too much.

Quote 256, HCP 8

### Health Care–Related Factors

#### Format of Professional Support

Considering the way support should be provided, 50% (8/16) of the HCPs mentioned the importance of frequency. Through frequent repetition of information, healthy behaviors by the patient, and reminders or feedback, a healthy lifestyle remains a topic of interest. However, the frequency of consultations in current practice is too low to do so. A total of 31% (5/16) of HCPs mentioned that support should be accessible at all times whenever the patient needs it. In addition, to provide tailored support, HCPs need data independent of the patients’ self-reports about their progress:

...we have tried to use a logbook, but a pedometer can track the walking process outside. You can respond to the objective information you receive. A logbook is just an estimate, you just have to believe that it’s true.

Quote 276, HCP 2

Most HCPs (10/16, 63%) found education as an important part of the intervention as patients lack knowledge or have misconceptions about their disease and a healthy lifestyle. Therefore, the health care system should play a role in offering trustworthy information, providing patients with concise pieces of information that are easy to understand:

...there is so much information available that they have no idea what to trust..., especially among the older population who have more respect for healthcare professionals, simple advice is really appreciated.

Quote 288, HCP 4

#### Relationship With the Patient

More than half of the HCPs (11/16, 69%) mentioned that their relationship with patients has a significant influence on the process. A good relationship helps in understanding the underlying reasons for patients’ behavior and motivation and creating a safe environment to share their feelings. Support does not end at the physical aspect of cardiac rehabilitation but entails mental support as well:

They suddenly are obliged to change a lot of things. I try to focus less on things that have to change, but acknowledge how it affects them...Therefrom, they will more easily comply with a lifestyle change in the end.

Quote 334, HCP 8

HCPs disagreed about their role as an authority figure. An equal relationship, in which they co-operated with their patients during the revalidation process, was frequently mentioned. However, 19% (3/16) of HCPs recognized that they function as the so-called *big stick* to keep patients on the right track.

#### Continuity of Professional Support

According to 63% (10/16) of HCPs, long-term support is crucial for maintaining a healthy lifestyle outside the health care environment. When the window of opportunity after a cardiovascular incident disappears, patients are more likely to return to old (unhealthy) habits. However, 44% (7/16) of HCPs mentioned lack of follow-ups or an end evaluation, leaving them with no ability to provide long-term feedback or information about the postrehabilitation success of the lifestyle intervention:

...when you want someone to follow through with the lifestyle change, you do have to check whether someone comprehends it and if is able to do so. When you let someone on their own, you will lose that person.

Quote 353, HCP 4

#### Organization of Care

All HCPs (16/16, 100%) mentioned at least 1 factor related to the way health care is organized, varying from physical facilities to nontangible influences. Most HCPs (11/16, 69%) mentioned a lack of financial resources and time. Furthermore, the limited co-operation between HCPs involved in cardiac care and those of other health care disciplines was mentioned as a barrier. There are few options for redirecting patients to another professional, disagreements within the extensive group of care providers, and insufficient information sharing between them:

The dietician for instance, she keeps her own records, she can’t attend the multidisciplinary team meeting due to her planning. I think that is a barrier as well, because we once had a patient who didn’t consume enough calories, which caused problems and that person did not feel well...[Quote 393, HCP 6]

More than half of the HCPs (7/16, 44%) mentioned a lack of attention for lifestyle within cardiac care or health care in general, although this varied greatly between domains (eg, cardiology and neurology). HCPs indicated that they missed regulations and protocols they could follow in providing lifestyle support.

### eHealth in Lifestyle Support

**Figure 1:**
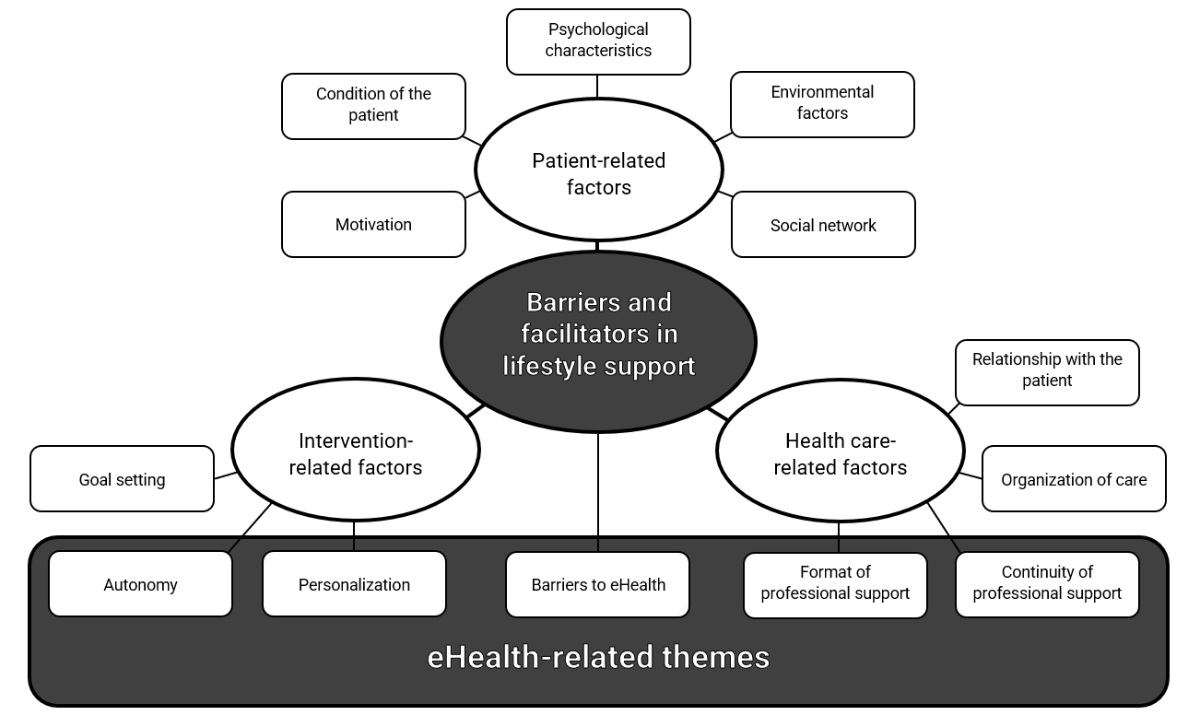
Overview of the identified barriers and facilitators in lifestyle support and their relation to eHealth.

Throughout the 12 themes discussed earlier, the use of eHealth reoccurred as a (potential) facilitator or solution to barriers, most prominently within the themes Autonomy, Personalization, Format of support, and Continuity of professional support (Figure 1). Although eHealth facilitators were more strongly related to lifestyle support, the barriers HCPs experienced were rather unrelated to lifestyle themes. To put more emphasis on this, the 13th theme concerning barriers in the implementation of eHealth will be discussed in the final part of this section (*Barriers to eHealth*).

### Benefits of eHealth

During analyses, a link between barriers to and facilitators of lifestyle support and potential facilitating benefits of eHealth emerged. The HCPs provided examples of how eHealth could help them.

#### Autonomy in eHealth

For patients to regain autonomy (theme 1), HCPs indicated a need for education and insight and saw an opportunity for eHealth to provide both. HCPs noticed that by giving patients the opportunity (and therefore the responsibility) of monitoring their own health through a digital tool, they can act whenever necessary (eg, adjusting their diet when they notice a higher blood pressure). According to HCPs, such insights would also enlarge awareness about unhealthy behavior and provide progress feedback to increase their motivation:

We offer cardiac patients who we follow-up via eHealth a pedometer, a digital scale that measures body fat percentages, a blood pressure monitor and a device to make an ECG. That provides them with insight into how they are doing.

Quote 107, HCP 14

Furthermore, as eHealth can be individually tailored, HCPs indicated that patients can control what information they receive and how they receive it. Patients can also work on their lifestyle at any time and in any way they wish to, increasing the level of self-management.

#### Personalization in eHealth

Personalization (theme 3) appeared to be key to lifestyle interventions, but HCPs raised the issue of identifying what patients wanted and needed. They thought that eHealth could help them to get more information about patients and their needs, before and during the intervention (eg, through web-based intake questionnaires). HCPs believed this could result in better adjustment of their support and more efficient consultations and lifestyle interventions:

In fact, even before someone comes in, you would have to start with: “This is the goal of the consultation.” Based on a test or questionnaire, you look at how someone can best be approached: What kind of advice do you prefer? There are probably apps, tools, questionnaires, and other things that can do so.

Quote 150, HCP 4

#### Format of Support in eHealth

Related to the format of support (theme 9), HCPs gave examples of how eHealth, especially its possibilities for remote support, could be beneficial. As digital tools are available at all times and not bound to a physical location, HCPs predicted that it would be much easier for patients to frequently have contact, work on their lifestyle, or receive information. This would also increase the accessibility of support:

That is the advantage of eHealth, that it is flexible, 24/7, which is really convenient. I think that a lot of people ruminate at night and would appreciate to write during nighttime. The possibility to do so at that very moment, not only when you meet your coach again. Then it has already faded away.

Quote 305, HCP 8

Consistent and automatic digital monitoring would provide more objective data, meaning HCPs would no longer have to rely on single measures during consultations or error-prone self-report measures. Furthermore, eHealth could be used as an educational platform, which HCPs thought they could use to provide patients with reliable and consistent information about their disease and lifestyle.

#### Continuity of Professional Support in eHealth

Continuity of professional support (theme 11) was mentioned as one of the biggest issues in current cardiac care. Therefore, most HCPs saw prolonged monitoring as a huge advantage of eHealth. Furthermore, this could enable HCPs to provide support in the long run, once patients return to their everyday lives:

There are gaps within the healthcare system, which makes it difficult for patients to continue independently. That is where this eCoach steps in. So during cardiac rehabilitation over here, they see the physiotherapist, they see the doctor, they can chat more easily through the portal.

Quote 359, HCP 6

### Barriers to eHealth

While recognizing these potential advantages of eHealth, HCPs raised some barriers concerning its adoption and usability. The most prominent concern of 63% (10/16) of HCPs was related to the general old age of patients with CVD, as older people are more likely to have little experience with or no interest in technology. Moreover, patients would generally prefer face-to-face contact over digital communication, either during the entire intervention or at least a part of it. One HCP explicitly mentioned the importance of face-to-face intake for a digital intervention to be successful:

They tell me: “Oh, I received a mail from online coaching, but I have already so much on my mind, so I just ignored it.”...But then they see me and say: “But now I know that it was you, that is nice!” It comes to life for them, in my experience at least.

Quote 466, HCP 8

Difficulties with technological tools and devices, such as bugs and slow development of the technology, were mentioned by 31% (5/16) of HCPs. In addition, they mentioned that there was no help desk for patients or HCPs. Furthermore, it was frequently mentioned by 31% (5/16) of HCPs that many current technological aids suffer from a low level of user-friendliness.

## Discussion

### Principal Findings

This study aimed to gain insights into the facilitators and barriers that HCPs experience in lifestyle support for the prevention and treatment of CVDs and investigate their views on potential eHealth tools. We interviewed 16 HCPs, resulting in 12 themes relevant to lifestyle support, of which four were related to eHealth. The 13th theme was related to eHealth barriers.

### Barriers to and Facilitators of Lifestyle Support

First, we aimed to identify the factors that HCPs find important in supporting patients with CVD in the uptake of and adherence to a healthy lifestyle. We found factors related to the intervention, patient, and health care system to help answer this question.

According to the HCPs, a lifestyle intervention should give patients a feeling of autonomy and possibilities for goal setting and allow for personalization. In line with our findings, in interview studies on lifestyle support for patients with diabetes, HCPs indicated that well-formulated goals create realistic patient expectations [15] and that standardized norms should be adjusted to patients’ capabilities [26]. Furthermore, HCPs indicated that it is no longer their role to tell patients to change their lifestyle but rather the patient’s responsibility [26]. However, although HCPs in this study named autonomy as an additional facilitator within lifestyle change, other studies reported patient responsibility to be a basic necessity because of low patient motivation [26] or even seem unrealistic as patients are not always able to independently start or maintain a healthy lifestyle [14].

With regard to patient factors, motivation to live healthily, the condition of the patient, psychological characteristics, environmental factors, and social networking were mentioned to be of influence within lifestyle support. HCPs working with people with (a high risk of) CVD [9,11,27], patients with diabetes [15,26,28], and chronic diseases in general [29] recognized similar factors, thereby suggesting that these are relevant within different patient populations. However, although HCPs in our study thought that little awareness of the impact of an unhealthy lifestyle on health contributed to a low level of patient motivation for change, a study with primary care HCPs reported that limited knowledge about risks of CVD is only seldom a barrier for engaging in lifestyle modification [14]. This discrepancy in results might be because of methodological differences, as the study by Jallinoja et al [14] included primary care HCPs and not HCPs mostly working in cardiac rehabilitation. Furthermore, primary care HCPs were asked about the relevance of insufficient knowledge to treatment and not lifestyle change per se. In addition, factors reported by patients with CVD themselves are relatively similar to those found in our study [37], which suggests that, at least in part, HCPs are able to recognize what patients need in lifestyle interventions.

Finally, several factors related to health care in general were mentioned, including the format of the provided support, continuity of professional support, the way care is organized, and the relationship between the HCP and patient. A high-quality relationship with the patient was also recognized as a facilitator within lifestyle support in other studies, as it would lead to both more collaborative patients and more motivated HCPs [30,34]. In addition, it would be easier to foster face-to-face encounters [30]. Similar to our results, the lack of time, little governmental responsibility, financial shortcomings, little co-operation between HCPs, and difficulties in referring patients were mentioned as barriers by HCPs involved in the prevention of CVD [11,16,27], type 2 diabetes [15,26], or in (chronic) diseases in general [29,38]. This shows that such barriers are not unique for lifestyle support in CVD rehabilitation, which provides HCPs and researchers with the opportunity to learn from other disciplines and work together to find solutions (eg, eHealth tools).

Other studies reported a lack of skills by HCPs to provide lifestyle support or a feeling that lifestyle interventions are ineffective as a barrier to the provision of lifestyle support [14,15,29,38]. These factors were not mentioned in this study, which might be owing to the nature of our sample that included HCPs who were specifically involved in lifestyle support and therefore might have a bigger skill set for and a more positive attitude toward providing lifestyle support.

### Barriers to and Facilitators of eHealth

Second, to determine what the (potential) facilitators of and barriers to eHealth tools would be in providing lifestyle support to patients with CVD, the interviewed HCPs described how eHealth could be applied to strengthen facilitators or solve barriers they encountered in lifestyle support. The statements that HCPs made concerning facilitators of eHealth were related to the intervention-related factors, Autonomy and Personalization. These advantages of eHealth have also been recognized by HCPs in other studies. Macdonald et al [34] reported that HCPs acknowledged that eHealth fosters the *two-way conversation*—a collaborative interaction between patients and HCPs, which explains why eHealth can create well-informed and autonomous patients. As HCPs previously indicated that lifestyle is the responsibility of the patient [11,19], eHealth could offer them tools that foster the patient’s autonomy. HCPs from other studies also indicated that eHealth helps them to personalize the program by getting to know their patients’ needs through the personal diary within the digital portal [39] and that personalization of an eHealth program is essential to fit the patient’s capabilities [40]. Furthermore, meta-analyses have demonstrated a positive relationship between both an autonomy-supportive health care climate and personalization of digital intervention content and successful behavior change [41,42].

With regard to health care–related factors, we found that the Format of professional support and the Continuity of support were important topics related to eHealth. Other studies have reported similar advantages of eHealth. Brandt et al [30] reported HCPs indicated that, because of its format, eHealth provides them with objective and measurable information and that it is not bound to a specific location or moment in time. In addition, some HCPs appreciated being able to follow-up their patients for a longer period, as it can be rewarding and increases their motivation and sense of responsibility to continue providing support [40]. Although we did not find a link between eHealth and the HCP-patient relationship, other studies have reported contradictory findings. Das et al [39] reported that eHealth does not have time constraints, shame, and fear of stigma, which leads to more self-disclosure from patients. However, Brandt et al [30] reported that HCPs indicated it is more challenging to establish an empathic relationship in a digital environment. This contrast might be because HCPs seem positive about tools that are an addition to face-to-face contact [39], but those that replace face-to-face interactions are perceived as less favorable to build a supportive relationship [30]. Furthermore, although we did not find the advantages of eHealth in the organization of care, other studies did. For example, other studies mentioned additional time by reusing old advice [30], co-operation between HCPs, and accessible alternatives to refer their patients to [32] as advantages of eHealth. Methodological differences related to the different care settings and organizational structures the interviewed HCPs worked in could explain this.

Despite the advantages that were recognized by HCPs from both our and other studies [30,32,34,39], there is a low level of acceptance and implementation of eHealth in health care [23]. HCPs in this study formulated several barriers that could offer an explanation. First, HCPs feel that because patients with CVD are older, they prefer face-to-face contact and have little technological experience; therefore, digital tools would not be suitable for this patient population. HCPs in another study made a distinction between current patients with CVD and future ones, as the latter will have substantially more experience in and affinity to technology [40]. In addition, the eHealth and face-to-face support preferences of patients with CVD vary greatly [43], which raises concerns about uneven eHealth adoption and unequal health benefits [44]. HCPs could possibly contribute to this, as the views and preferences of patients are important in their decision to use eHealth [40]. At the same time, Grünloh et al [45] suggested that some HCPs seem to be unaware of the development of patient skills and knowledge over time. This could mean that once HCPs believe a patient is a technology-averse person, there will be minimal attempts to help the patient become acquainted with eHealth. eHealth acceptance could also be influenced by preference for face-to-face communication of HCPs themselves [30,32], which could be because of concerns regarding the therapeutic alliance with their patients [40]. However, others do not experience this issue, as they use eHealth for information sharing (eg, educational texts) rather than communication purposes (eg, interacting with patients) [31].

Other barriers identified concerned eHealth apps themselves, such as bugs and the slow development of digital tools, the lack of a help desk, and a low level of user-friendliness for both patients and HCPs. Other studies mentioned similar concerns, such as limited innovation, being offered digital tools that were still under development, and digital information that is too difficult to interpret and translate into support for their patients [33,34]. A study on an eHealth tool evaluation showed that, in hindsight, HCPs have specific wishes concerning the utility and design of such tools [32]. If they were included in the development process at an early stage, such barriers could have been prevented [21,24].

In this study, barriers to eHealth were not related to organizational factors. However, HCPs have previously shown concern about the inflexibility of the health care system and indicated that organizational structures and attitudes of HCPs have a major impact on eHealth acceptance and implementation [21,39,40,44]. The lack of financial compensation also played a role according to HCPs from previous studies [21,22]. Therefore, apart from barriers experienced by individual HCPs, overcoming structural obstacles seems necessary for the implementation of eHealth in health care [46]. As many HCPs in our sample were already working with eHealth tools, they might have experienced fewer organizational difficulties and, therefore, did not mention such organizational barriers.

To make eHealth implementation more successful in practice, the results of this study suggest that HCPs do not need to be convinced about the benefits of eHealth but rather that the barriers they experience should be resolved. To overcome these barriers, health policies could play an important role in the provision of support and equipment. This way, HCPs would be able to implement the reported benefits of eHealth in lifestyle support for people with CVD.

### Limitations and Future Studies

First, our results were based on the opinions and interpretations of HCPs and not on the actual views of the patients themselves, who might have an alternative view on how eHealth can support them. Future studies could therefore conduct interviews with both HCPs and their patients to compare their views and attitudes toward lifestyle support and the use of eHealth.

Although we intentionally interviewed health care HCPs involved in the lifestyle support of patients with CVD, this specific sample limits the generalizability of our results as our sample has experience with and might be more willing to provide lifestyle support, whereas other HCPs might be less inclined to. It would therefore be interesting to investigate how different levels of experiences with and attitudes toward lifestyle support and eHealth translate into differences in the barriers experienced by HCPs.

Finally, we did not explicitly ask about the association between facilitators of and barriers to lifestyle support and the use of eHealth as a possible solution. Future studies should therefore investigate how eHealth can help overcome barriers related to specific aspects of lifestyle support experienced in various health care settings. These results could provide eHealth developers with a better direction in the development of eHealth interventions.

### Conclusions

This study provides insights into Dutch HCPs’ views on lifestyle support and eHealth in cardiac care. We identified facilitators and barriers related to intervention-, patient-, and health care–related factors. HCPs in general showed high approval of lifestyle support for patients with CVD and identified the potential benefits of incorporating eHealth. However, the interviews also revealed several barriers that impede HCPs’ use of eHealth in lifestyle support. Incorporating their needs and values in the development of lifestyle support programs, especially eHealth, could increase their use and lead to a more widespread adoption of eHealth into health care.

## Abbreviations

CVD: cardiovascular disease
HCP: health care professional
